# Functional effects of proinflammatory factors present in Sjögren’s syndrome salivary microenvironment in an *in vitro* model of human salivary gland

**DOI:** 10.1038/s41598-017-12282-x

**Published:** 2017-09-19

**Authors:** Mayte Arce-Franco, María Dominguez-Luis, Martina K. Pec, Carlos Martínez-Gimeno, Pablo Miranda, Diego Alvarez de la Rosa, Teresa Giraldez, José María García-Verdugo, José David Machado, Federico Díaz-González

**Affiliations:** 1 Servicio de Reumatología, Hospital Universitario de Canarias, Tenerife, Spain; 2 Servicio de Cirugía Máxilofacial, Hospital Universitario de Canarias, Tenerife, Spain; 3 Departamento de Ciencias Médicas Básicas and Instituto de Tecnologías Biomédicas, Universidad de La Laguna, Tenerife, Spain; 4 Laboratorio de Neurobiología Comparada, Instituto Cavanilles, Universidad de Valencia, Valencia, Spain; 5 Departamento de Medicina Física y Farmacología, Universidad de La Laguna, Tenerife, Spain; 6 Departamento de Medicina Interna, Dermatología y Psiquiatría, Universidad de La Laguna, Tenerife, Spain

## Abstract

Primary Sjögren’s syndrome (pSS) is an autoimmune exocrinopathy in which the role that the immune response plays in reducing exocrine gland function, including the glandular microenvironment of cytokines, has not been fully understood. Epithelial cells from biopsies of human parotid gland (HPG) were used to establish a model of human salivary gland *in vitro*. In this model, the functional consequences of several proinflammatory soluble factors present in the pSS glandular microenvironment were assessed. Stimulation with isoproterenol and calcium produced a significant increase in the basal activity of amylase in the HPG cell supernatants. Under these conditions, the presence of TNF-α and CXCL12 increased amylase mRNA cellular abundance, but reduced the amylase activity in the cell-free supernatant in a dose-dependent manner. IL-1β and IFN-γ, but not TGF-β, also diminished amylase secretion by HPG cells. These results suggest that the glandular microenvironment of cytokine, by acting post-transcriptionally, may be responsible, at least in part, for the reduced exocrine function observed in pSS patients. These data may help to a better understanding of the pathogenesis of SS, which in turn would facilitate the identification of new therapeutic targets for this disorder.

## Introduction

Primary Sjögren’s Syndrome (pSS) is a chronic autoimmune disorder of unknown etiology characterized by lymphocytic infiltration of exocrine glands, both salivary and lachrymal, that results in dry eyes and mouth^1^. In addition to characteristic sicca syndrome, most patients with pSS suffer extraglandular manifestations; including a fourteen fold increased risk for developing non-Hodgkin B-cell lymphoma than the general population^2^.

pSS is one of the most common autoimmune diseases, with prevalence rates that can vary depending on the classification criteria used from 0.09^3^ to 0.72%^4^ and a clear preponderance of women versus men, 10:1^5^. A number of classification criteria for pSS have been developed, all of them based mainly on histological findings of minor salivary glands biopsies^6–8^. The pathologic hallmarks of pSS are: 1) the presence of focal lymphocytic aggregates that results in the loss of tissue architecture; and 2) atrophic involution of the acini. In contrast to the traditional conception supporting that symptoms of this disease result from structural damage of the glandular tissue^9,10^ two observations indicate that this view is too simplistic: 1) xerostomia may precede the development of relevant acinar atrophy in pSS^11,12^, and 2) acinar atrophy is a physiological mechanism related to age itself^13^, while salivary glands maintain a large functional reserve allowing a normal flow of saliva even at advanced ages^14^. This evidence strongly suggests that functional impairment of glandular tissue may play a more relevant role than the structural damage in the pathogenesis of pSS.

The inflammatory infiltrate present in exocrine glands of pSS patients is constituted by activated T lymphocytes, mainly CD4+, B cells and dendritic cells^15^. Quantitative real-time PCR (qRT-PCR) studies have demonstrated that CD4+ T lymphocytes obtained from salivary gland biopsies of pSS patients express higher amounts of IL-2, IFN-γ, and IL-10 mRNA than peripheral blood CD4+ T cells, and salivary gland epithelial cells produced more IL-1, IL-6, and TNF-α mRNA than epithelial cells in normal salivary glands^16^. Quantification of these cytokines by ELISA in saliva of patients respect to controls showed an elevated concentration that correlated with the mRNA levels^16^. Similar results have been obtained by *in situ* hybridization technique^17^. On the other hand, ectopic production of the lymphoid chemokines CXCL13, CCL21 and CXCL12^18,19^ has been shown to correlate with the severity of sicca syndrome^20^, as well as with lymphoid organization in pSS patients^18^. Although all these data suggest that cytokines and chemokines play a central role in pSS’s pathogenesis, the real implication of these proinflammatory soluble factors on exocrine gland dysfunction in this syndrome is still poorly understood.

In the present study, we have established an *in vitro* model of human salivary gland suitable for functional studies. Our data demonstrate that the presence of TNF-α, IL-1β, IFN-γ and CXCL12 reduces exocrine capacity in human glandular epithelial cell lines at a post-transcriptional level. This data may lead to a better understanding of the pathogenesis of pSS, which in turn would facilitate the identification of new therapeutic targets for this disorder.

## Results

### Morphology and proliferation of primary culture cells from human salivary glands

**Figure 1:**
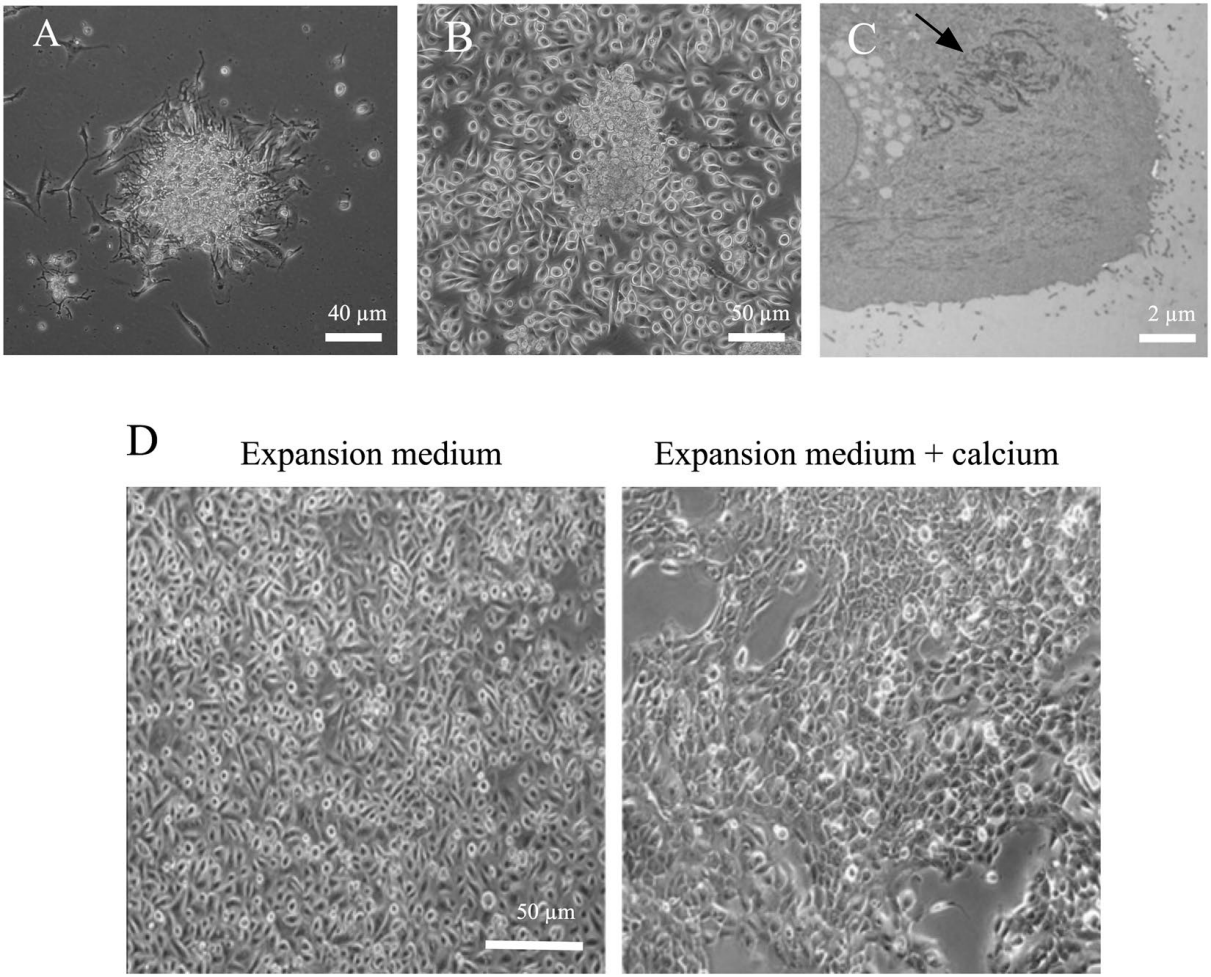
Morphology of HPG in culture. (**A**) Phase-contrast image of a typical HPG explant (around 100 cells/colony) cultured *in vitro* (10x) 24 h after seeding. (**B**) Phase-contrast image of HPG epithelial cells forming a monolayer (confluence of 90%) after a month of culture. (**C**) Picture showing intracellular keratin filaments (indicated by the arrow). A representative image is shown. (**D**) Phase-contrast images (10X) showing the morphology of HPG cells cultured in expansion medium (without calcium) and in expansion medium with 2 mM calcium for 24 h.

Human parotid glands (HPG) primary cells were isolated from tissue samples and expanded in culture using expansion medium, as described in Methods section. Figure 1A shows a representative micrograph of HPG cells forming a colony of epithelial-shaped cells after two days of seeding. After one month in culture, cells were mostly polygonal and had formed an epithelial-like monolayer (Fig. 1B). Under these culture conditions, the duplication time of HPG cells, as assessed by the DNA content, was approximately 7 days. Electron microscopy images confirmed the presence of keratin filaments (Fig. 1C), structural proteins characteristic of epithelial cells^21^.

When HPG cells were cultured for 24 h in expansion media containing 2 mM CaCl_2_, cells changed their basal morphology increasing cell-cell contacts. The original cell morphology was recovered again after 24 h of culture in low calcium media (Fig. 1D).

These data indicate that HPG cells growth in culture displayed an epithelial morphology.

### Primary culture HPG cells retain the capacity for β-adrenergic agonists-induced amylase secretion

**Figure 2:**
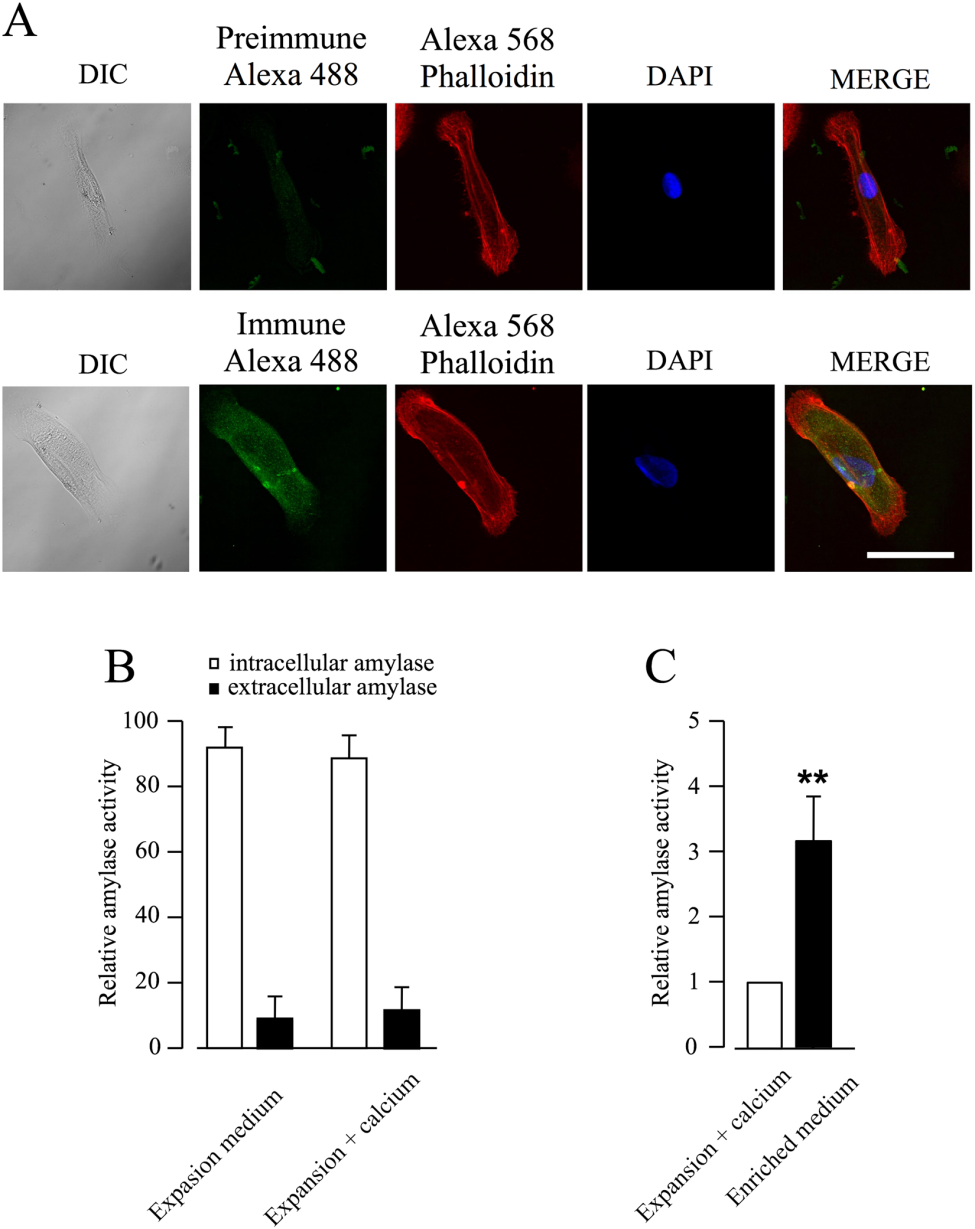
Released amylase activity of HPG cells in culture. (**A**) Confocal images showing the differential interference contrast images (DIC), amylase immunoreactive (Alexa Fluor 488), the presence of intracellular actin filaments (Alexa Fluor 568 phalloidin), cell nucleous (DAPI) and a merged image in cultured HPG cells. (**B**) Bar graph showing the relative intracellular versus extracellular amylase activity in cells incubated with either expansion medium without or with 2 mM calcium. Data represent the relative mean ± SEM from eight independent experiments. (**C**) Bar graph showing amylase activity in the cell-free supernatant between cells cultures in expansion medium with 2 mM calcium or in enriched medium (expansion medium with 2 mM calcium and isoproterenol 10 µM). Data represent the relative mean ± SEM from eight independent experiments with respect to cells cultured in expansion medium plus calcium, which was considered 1. **p ≤ 0.01 by Wilcoxon signed-rank test.

Figure 2A shows that, under our experimental conditions, HPG primary cells contain intracellular immunoreactive amylase. Intracellular amylase content proved to be approximately 9 times greater than the extracellular amount, independently of extracellular Ca^2+^ presence (Fig. 2B), indicating a slight constitutive amylase basal release in culture HPG cells.

To study the secretory capability of cultured HPG cells, we measured amylase activity in cell supernatants incubated in expansion medium containing 2 mM Ca^2+^ or in enriched medium (containing 2 mM Ca^2+^ and 10 µM isoproterenol, a β-adrenergic agonist) for 18 h at 37 °C. The presence of Ca^2+^ and isoproterenol led to a three-fold increase in amylase activity in cell-free supernatants, demonstrating that secretion of this enzyme is regulated in HPG cells (Fig. 2C).

These data indicate that HPG primary cells cultured under our experimental conditions contain amylase and, most importantly, that these cells are able to release it in response to β-adrenergic stimulus.

### Characterization of primary culture HPG cells

**Figure 3:**
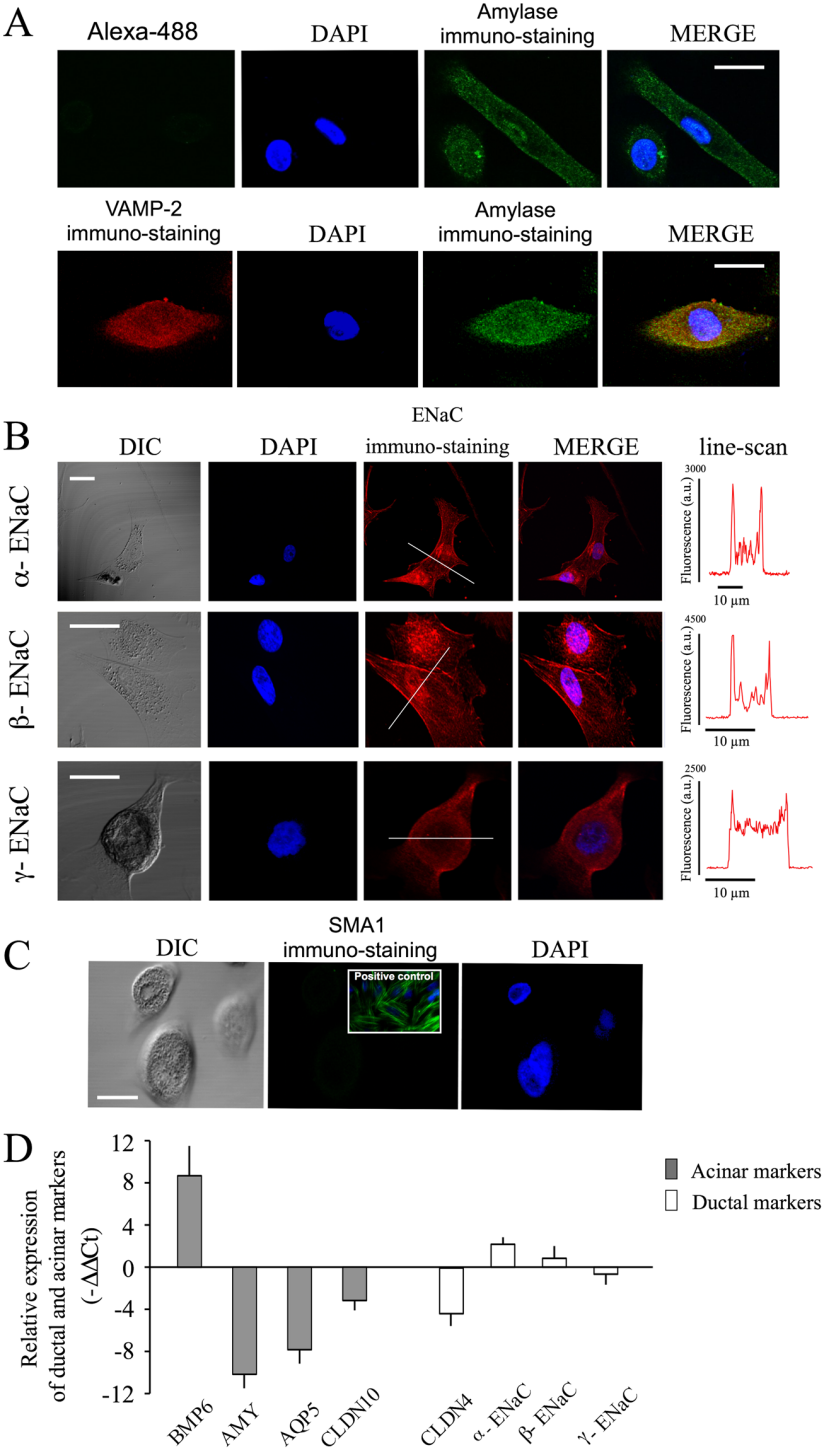
Expression pattern of endogenous amylase and ENaC subunits in HPG cells. (**A**) Series of confocal images (x-y midsections) showing the expression pattern for α-amylase and VAMP-2, as well as DAPI and merged images. (**B**) Representative images showing the distribution of α−, β−, and γ−ENaC subunits in HPG cells. DIC images, DAPI and merged are also shown. Scan-line shows the fluorescence intensity profile, suggesting the presence of ENaC in the plasma membrane. (**C**) Confocal images showing the absence of myoepithelial cells in the culture by SMA1 staining. Inset shows the positive control of SAM1 staining in culture rat vascular smooth muscle cells. Scale bar 10 µm. (**D**) Quantitative RT-PCR from parotid gland biopsy and from parotid gland cells in culture were carried out (∆∆Ct method). Graph showing the expression differential level of specific acinar (bone morphogenetic protein 6 -BMP6-, aquaporin-5 -AQP5-, amylase -AMY- and claudin 10 -CLDN10-) or ductal (claudin 4 -CLDN4- and ENaC Channel (α-, β- and γ-)) genes from cultured cells respect to biopsies of they come from. Data are a representative experiment out of four.

Human salivary glands are composed of acinar, myoepithelial, and ductal epithelial cells. In order to characterize cell types present in HPG cell culture, specific antibodies were used. The expression of amylase, a serous acinar cell marker was compared with the distribution of VAMP-2, a major and constitutive component of secretory vesicles from neurons and neuroendocrine cells^22^. The presence of immunoreactive α-amylase was detected in almost all HPG cells. Confocal microscopy images show endogenous amylase with the characteristic punctuated distribution partially co-localized with VAMP-2 (~20 ± 4%, mean ± SEM; n** = **189 randomly taken spots from 12 randomly selected cells (Fig. 3A).

The expression of α-, β- and γ-ENaC subunits, sodium channels present in the apical membranes of many tight epithelia^23^ was used to detect the presence of ductal epithelial cells in HPG cultures. Confocal images showed plasma membrane-associated staining of the three subunits, indicating the presence of cells with ductal characteristics (Fig. 3B). All cells were negative for SMA1, a myoepithelial cell marker (Fig. 3C).

To further characterize these primary cell lines, we studied the relative differential expression of acinar or ductal markers at mRNA level between frozen gland biopsy samples and the cell lines that they generated, after a month in culture. The expression level of the specific acinar marker BMP6 was higher in cultured cells than in gland tissue (Fig. 3D), suggesting an enrichment of acinar cells in culture. However, expression levels of AQP5, amylase and CLDN10, which are also specific to acinar cells, were lower in the cultured cells than in the tissues. We also detected the expression of CLDN4 and the α-, β- and γ-ENaC subunits, which are specific markers of ductal cells. Whereas the expression of CLDN4 was lower in HPG cultured cells than in tissues, the expression of α-, β-ENaC channels subunits was higher (Fig. 3D).

Taken together, these data show that after a month in culture HPG cells are amylase-producing cells that display characteristics of both acinar and ductal cells.

### HPG cells express functionally active surface ENaC at the membrane

**Figure 4:**
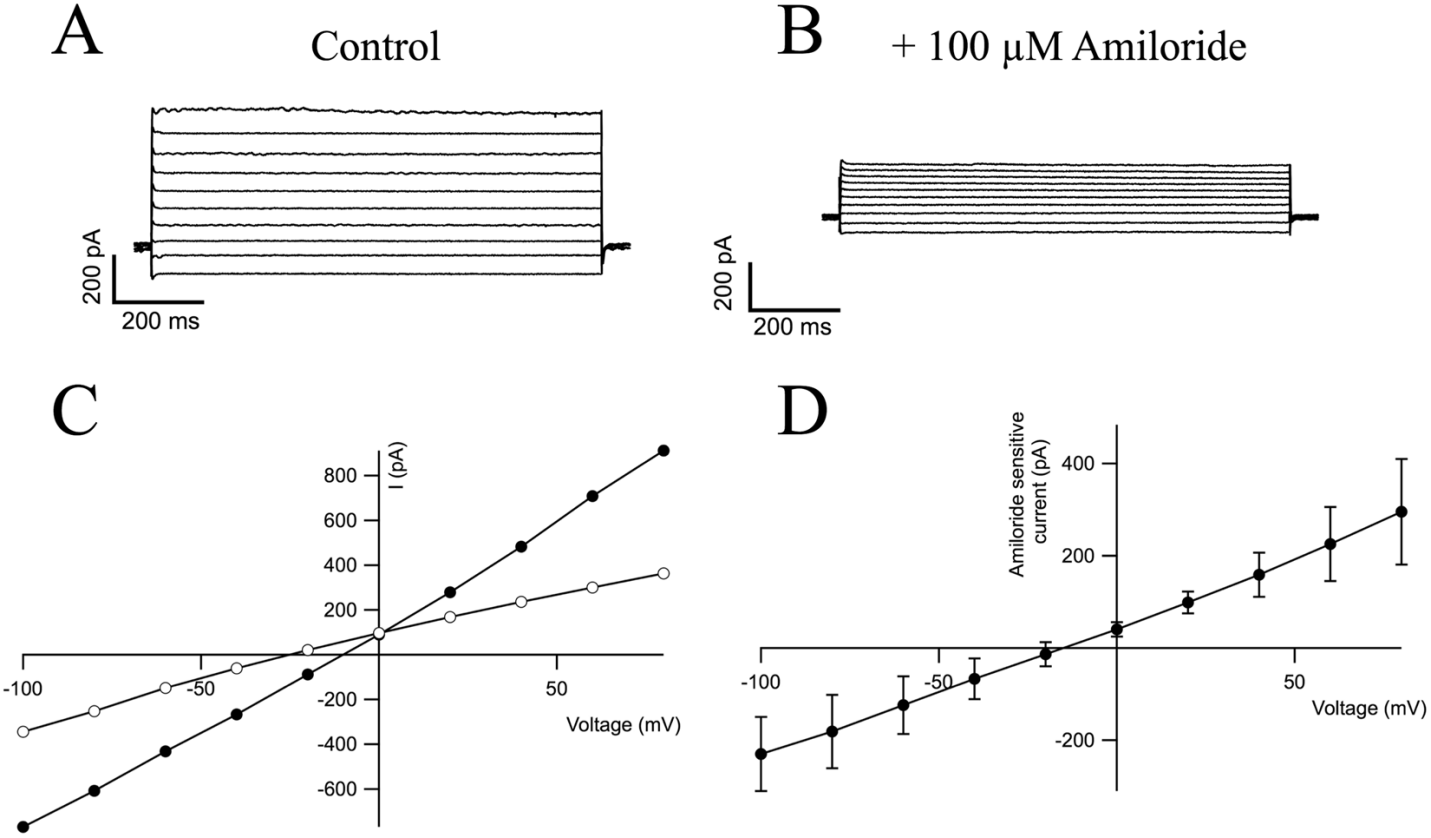
HPG Patch clamp. (**A**) HPG cells express functional ENaC channels at the plasma membrane; representative whole cell ionic currents recorded with various depolarizing pulses from a holding potential of −70 mV in the absence of amiloride (control). (**B**) Representative whole cell ionic currents recorded with various depolarizing pulses from a holding potential of −70 mV after the addition of 100 µM amiloride. (**C**) I-V curves before (black circles) and after the addition of amiloride (open circles). (**D**) I-V subtraction curve showing the amiloride-sensitive component. Bars represent SEM, n = 5.

HPG cell lines express the three subunits of ENaC at the plasma membrane (Fig. 3B). The functionally of this ion channel in HPG cell lines was studied by patch-clamp technique. Figure 4A shows the currents elicited from a holding potential of −70 mV by voltage steps in a HPG cell in the perforated-patch configuration. The currents promptly reached steady-state following each voltage step and did not show any significant voltage-dependent activation or inactivation. Following replacement of the NaCl solution in the bath with a solution containing *N*-methyl-D-glucamine, the currents recorded at negative pipette potentials were markedly reduced (data not shown). ENaC currents can be identified as amiloride-sensitive Na^+^ currents (Fig. 4B), as described previously^24^. Amiloride-sensitive whole-cell currents were observed in all cells studied, yielding an average value of 225 ± 80.5pA (Fig. 4C). The average steady-state current-voltage (I-V) relation from the five experiments is shown in Fig. 4D.

These experiments with whole-cell recordings demonstrate that ENaC channels are functionally expressed in the membrane of HPG cell lines.

### Effect of cytokines and chemokines on β-adrenergic-induced amylase secretion by HPG cells

**Figure 5:**
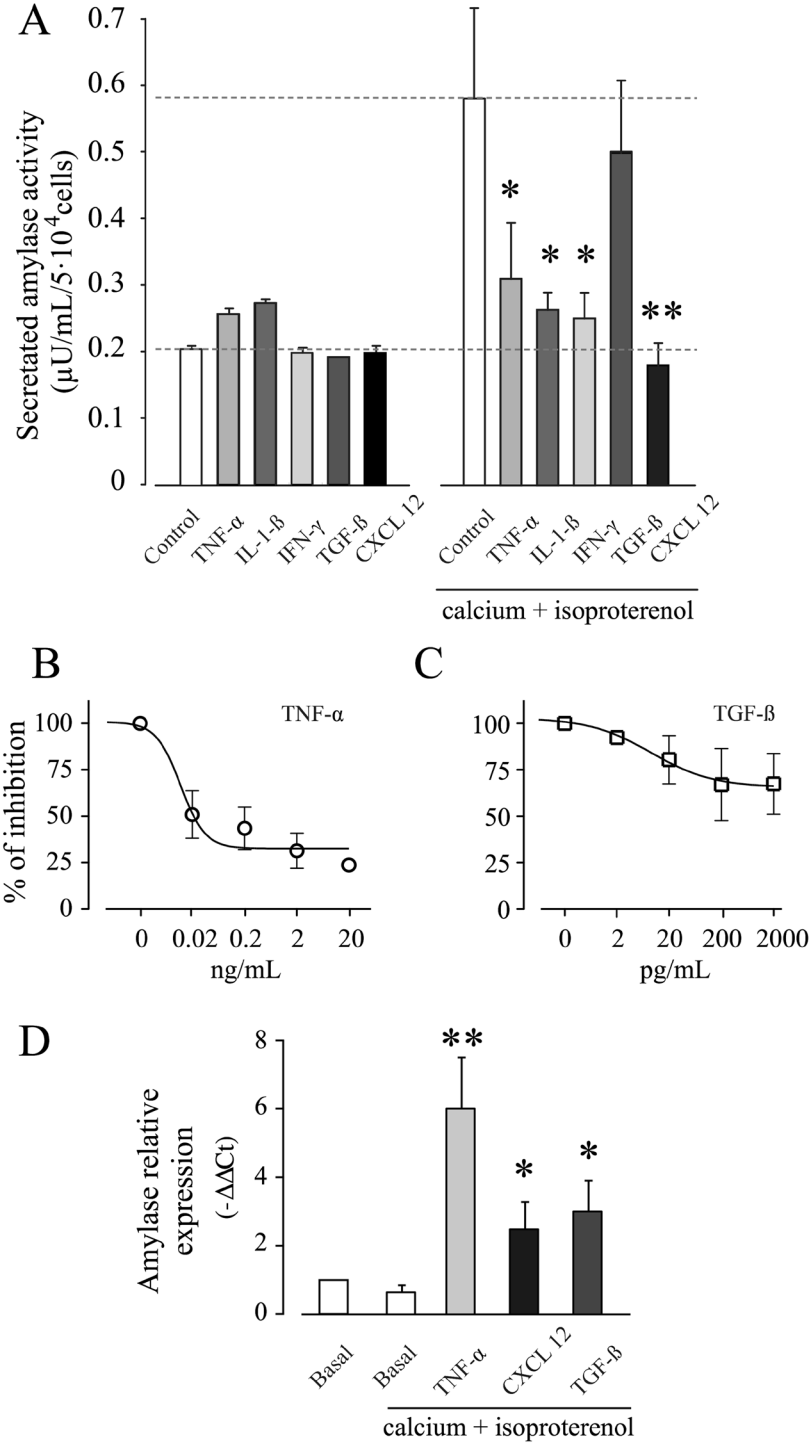
Functional effect of cytokines and chemokines on the release of amylase activity and amylase mRNA content in HPG cell lines. (**A**) Amylase secretion assay from HPG cells under resting conditions and after stimulation with the ß-adrenergic agonist isoproterenol (10 µM) and calcium (2 mM) in the presence of: TFN-α (20 ng/mL), IL1-ß (5 ng/mL), IFN-γ (100 ng/mL), TGF-β (2 ng/mL) and CXCL12 (100 ng/mL). *p ≤ 0.05 and **p ≤ 0.01 by Wilcoxon signed-rank test respect to control. (**B**) Dose–response curves showing the inhibition of amylase secretion in presence of TFN-α. The solid line represents the sigmoidal fit to the data. The IC50 calculated for TFN-α was ~0.1 ng/mL. A representative experiment out of three is shown. The amylase activity in the absence of soluble factor was considered 100%. IC50 values were determined by variable-slope sigmoid function using GraphPad Prism version 5.04 for Windows (GraphPad Software, San Diego CA). (**C**) Dose–response curves showing the effect of TGF-β on amylase secretion. A representative experiment out of three is shown. The amylase activity in the absence of soluble factor was considered 100%. (**D**) Bar graph showing the relative amylase mRNA content in HPG cells cultured for 24 h in the presence of 20 ng/mL TNF-α, 100 pg/mL CXCL12 or 2 ng/mL TGF-β with respect to cells maintained in medium containing calcium (2 mM) and isoproterenol (10 µM). Data represent the mean ± SEM of 3 independent experiments. *p ≤ 0.05 and **p ≤ 0.01 by Wilcoxon signed-rank test.

To study whether a proinflammatory microenvironment alters the secretory ability of HPG cells, amylase activity was measured in cell-free supernatant of cells incubated with expansion medium (without Ca^2+^) or enriched medium (2 mM Ca^2+^ plus 10 μM isoproterenol) in the absence or presence of different cytokines and chemokine (Fig. 5A). Non-exocytotic, constitutive release or basal amylase activity in the supernatant of HPG cells was 0.2 ± 0.07 µU/mL/5 × 10^4^ cells, a quantity which increased significantly (3-fold) in the presence of 2 mM Ca^2+^ and 10 μM of isoproterenol (Fig. 5A). After 24 h incubation expansion medium in the presence of 20 ng/mL TNF-α, 5 ng/mL IL-1β, 100 ng/mL IFN-γ, 2 ng/mL TGF-β or 100 pg/mL CXCL12, basal amylase activity in the cell-free supernatant did not change. However, when HPG cells were incubated under the same conditions in enriched medium, TNF-α, IL-1β, IFN-γ and CXCL12, but not TGF-β, caused a significant reduction in cell-free supernatant amylase activity (Fig. 5A). Figure 5B shows that after 24 h of culture, TNF-α induced a dose-dependent reduction in amylase activity in cell-free supernatants with a maximum effect of TNF-α at 2 ng/mL (IC50~0.1 ng/mL). A similar dose-response was obtained with CXCL12 (IC50~30 pg/mL, with a maximum response at 1 ng/mL) (data not shown). The presence of TGF-β did not significantly modify amylase activity in supernatants (Fig. 5C).

To investigate the relation between secreted amylase and expression of the gene, we measured the content of amylase mRNA transcripts by qRT-PCR in HPG cells culture in either expansion medium or enriched medium in the absence or presence of 20 ng/mL TNF-α, 100 pg/ml CXCL12 or 2 ng/mL TGF-β. Cells treated with TNF-α, CXCL12 and TGF-β showed a significant increase of amylase mRNA content with respect to basal levels (Fig. 5D), demonstrating that decreased secretion occurs post-translationally and is not a consequence of diminished amylase mRNA abundance.

## Discussion

The major findings of this study are as follows: 1) The primary cell lines obtained from human parotid gland biopsies show in culture characteristics of both acinar and ductal cells; 2) these cell lines are suitable for functional studies as they retain the capacity for β−adrenergic agonist-induced amylase secretion; and 3) interestingly, TNF-α, IFN-γ, IL-1β and CXCL12 reduce amylase secretion in these cell lines at the post-transcriptional level, suggesting that the cytokine microenvironment present in glands of pSS patients might be responsible, at least in part, for the reduced exocrine function that characterizes this syndrome.

pSS glandular lymphocytic infiltrate contains T and B cells, which in association with resident epithelial cells, generate a proinflammatory microenvironment containing IL-1, IL-6, TNF-α and IFN-γ^16,17^ among other cytokines^25–27^, a number of chemokines^18,19^, as well as growth factors, such as nerve growth factor-β^28^. It has been suggested that the mechanism for gland destruction in pSS is the apoptosis of ductal cells. Different mechanisms, including cytotoxic effector actions of T lymphocytes^29^, as well as the presence of IFN-γ^30^ and IL-17^25^, have been implicated in causing apoptosis in secretory gland cells. However, in pSS the overall glandular architecture is preserved, and the reduction in exocrine gland function does not seem it can be simply explained by acinar atrophy^11–13^.

The role that the proinflammatory microenvironment present in the glandular tissues plays in the pathogenesis of pSS remains poorly studied, probably due to lack of *in vitro* models suitable for functional studies. The use of primary cells represents the best option for creating an artificial salivary gland^31^ where to study the physiopathology of processes that affect the salivary production. In this study, we have developed an experimental culture protocol based on that previously described by Chopra *et al*.^32^ that allows to isolate and expand primary epithelial cells from human parotid gland biopsies. After enzymatic dispersion, HPG primary cells adhered to collagen-forming colonies of epithelial-shaped cells. An ultrastructure study of these cells revealed the presence of keratin filaments, a typical finding of epithelial cells^21^. Cells responded to the presence of calcium in the extracellular medium, increasing their cell-cell contacts. This suggests the formation of tight junctions among cells, similar to what has previously been described^33^.

With regard to gene expression, we found significant changes in the expression profiles of genes typically associated with acinar and ductal cell phenotype, with both enrichment and repression of specific markers of both cell types. The expression of BMP6 mRNA was higher in HPG cultured cells than in glandular tissue from which they came from. This circumstance is similar to that observed in glandular epithelium of patients with pSS where the expression of BMP6 mRNA is significantly increased respect to controls^34^. It has been reported that BMP6 inhibits AQP5 expression, an effect that has been related to decreased saliva production in a mice model of pSS^35^. In our model, AQP5 was consistently downregulated in HPG cells with respect to the whole glandular tissue from which they came. It has been reported that the expression pattern of claudins changes with culturing and this may impact the ability of the cells to function as acinar cells^36^. In our model, we found decreased expression of CLDN10, AQP5, and amylase, changes that suggest a partially dedifferentiated acinar phenotype. A similar effect has been previously described in rat parotid cell cultures^36^. ENaC is a heteromultimeric, voltage-independent, amiloride-sensitive, ion channel^37^ expressed in the apical membrane of salivary gland ducts, where they play a key role in ductal Na^+^ reabsorption, and producing hypotonic saliva^38^. Functional expression of ENaC has been demonstrated at the single cell level by whole-cell recordings in intralobular duct cells isolated from the mouse submandibular gland^39^. Notably, we found expression, membrane targeting and functionally capable ENaC in an HPG primary cell line. The assessment of changes in gene expression in HPG cell line suggests that, under our experimental condition, this cell line in culture dedifferentiate and display intermediate characteristics of both acinar and ductal cells.

Amylase, the major protein in saliva, is released by the salivary gland mainly through the intracellular cyclic AMP pathway via sympathetic activity (ß-adrenoceptor stimulation)^40^. Assessments of amylase have been used as surrogate markers of saliva production in different experimental approaches both *in vitro* and *in vivo* ^32,41,42^. Immunofluorescence experiments detected immunoreactive amylase in the typically punctuated distribution involving partial localization with the VAMP2, similarly to what has been described in primary cultures of rat parotid cells^43^. This indicated that amylase was partially stored in the secretory vesicles of HPG cells. When these cells were exposed to media containing calcium and isoproterenol, 50% of the total amylase contained in secretory granules (20% of the total immunoreactive amylase) was released, results that suggest that exocytosis is a regulated process in HPG cells. Consequently, our experimental model represents an *in vitro* model of the human salivary gland suitable for functional studies.

Most of the research on the roles that cytokines and chemokines play in pSS pathogenesis has focused on their apoptotic effects on epithelial cells^25,30^, on their modulatory effect on infiltrating lymphocyte subpopulations^44^, on salivary gland lymphoid organization^19,45^, or on their role in inducing pSS-associated lymphoma^46^. However, little attention has been placed on the potential functional effect that the cytokines and chemokines present in the glandular microenvironment of pSS patients exert on the exocrine function of the salivary gland. In this work we have studied soluble factors present in the glandular microenvironment which have been related with the exocrine dysfunction in pSS patients^16–18^. When HPG cells were incubated in medium containing calcium and isoproterenol, the presence of TNF-α, IL-1β, IFN-γ and CXCL12 caused a significant reduction in amylase activity in the cell-free supernatant with respect to basal conditions. However, TGF-β, a cytokine with anti-inflammatory/profibrotic effects^47^, did not modify the basal extracellular amylase activity induced by calcium and isoproterenol in HPG cells. The content of amylase mRNA transcripts assessed by qRT-PCR in HPG cell cultures under the same conditions showed that treatment with TNF-α, and to a lesser degree, CXCL12 and TGF-β, showed a significant increase in amylase mRNA content with respect to basal levels. Supporting this finding, previous data have shown that TNF-α and, to minor degree TGF-β, activate the amylase promoter in a human cell line of the submandibular salivary gland^48^. The molecular mechanisms involved in the inhibition of amylase secretion by cytokines in our cell system were not explored. However, taking into account that these effects were observed after 24 h, it is very likely that it could be related with downregulation of proteins involved in the regulated exocytosis. In this regard, in human salivary gland acinar cells, TNF-α treatment down-regulates the expression of AQP5, a water channel protein required for salivary secretion^35,49^.

In summary, the data presented herein suggest that TNF-α, IL-1, IFN-γ and CXCL12 are capable of interfering *in vitro* with the exocrine function of epithelial cells from the human salivary gland. Since these soluble factors are present in the glandular microenvironment of pSS patients, it is possible that they might participate in the pathogenesis of this disorder in a manner different to what has been previously thought; i.e., interfering with ability of glandular epithelial cells to produce saliva.

## Methods

### Antibodies and reactives

The following antibodies were used: anti-vesicle-associated membrane protein (VAMP)-2 mouse monoclonal antibody (mAb), purchased from Abcam (Boston, MA); anti-human smooth muscle actin (SMA1) mouse mAb from Sigma-Aldrich (St Louis, MO); anti-rat epithelial sodium channel (ENaC) α−, β− or γ−subunits rabbit polyAbs with human cross reactivity kindly provided by Dr. Cecilia Canessa (Yale University)^50^; and anti-human amylase rabbit polyAb and mouse monoclonal, purchased from Abcam. Alexa Fluor 568 phalloidin 568 was obtained from Molecular Probes (Invitrogen). Secondary antibodies were goat anti-mouse or anti-rabbit polyAbs conjugated to Alexa Fluor 568 or Alexa Fluor 488 (Molecular Probes), respectively.

Collagen A, L-glutamine, penicillin and streptomycin were purchased from Biochrom (Berlin, Germany). Collagenase P, cOmplete protease inhibitor and insulin were obtained from Roche Diagnostics (Mannheim, Germany). CXCL12 was purchased from (R&D Systems, MN) and TNF-α, IL-1, IFN-γ and TGF-β from Sigma Aldrich. Bovine serum albumin (BSA), dimethyl sulfoxide (DMSO), epidermal growth factor (EGF), 4-(2-hydroxyethyl)-1-piperazineethanesulfonic acid (HEPES), hydrocortisone, isoproterenol, *N*-methyl-D-glucamine, nystatin, phenylmethylsulfonyl fluoride (PMSF), and Triton X-100 were also obtained from Sigma-Aldrich. Glutaraldehyde and paraformaldehyde were purchased from Panreac (Barcelona, Spain).

T25 flask and 96 well-plates were acquired from Corning (Corning, NY). MCDB 153 basal medium (Ca^2+^ <4 × 10^−5^ M) was obtained from Biochrom.

### Human samples, cell isolation and culture

Samples of the parotid gland were obtained from the biopsies of patients undergoing non-oncological maxillofacial surgery. All procedures were performed in accordance with institutional and national regulations and written informed consent was obtained from each patient before tissue collection. The final version of the protocol was approved by the Ethic Committee of Hospital Universitario de Canarias.

Primary human parotid gland (HPG) cell lines were cultured in a manner similar to that described by Chopra^32^. Briefly, glandular tissue was cut using fine forceps and scissors, washed with phosphate-buffered saline without calcium and magnesium (PBS) and incubated in MCDB 153 basal medium supplemented with 10 IU/mL penicillin, 0.1 μg/mL streptomycin and with 0.05% bacterial collagenase P for 20 minutes at 37 °C with slow stirring. Then cell were pelleted, washed twice in PBS, plated on T25 flasks (pre-coated with 5% collagen A for 1 h at 37 °C) and cultures in expansion medium: MCDB 153 basal medium, 1% HEPES, 50 IU/mL penicillin, 50 μg/mL streptomycin, 2 M L-glutamine, 10 μg/mL insulin, 500 μg/mL hydrocortisone and 10 μg/mL EGF. Cells were maintained in a humidified atmosphere of 5% CO_2_ at 37 °C. After confluence, cells were harvested with trypsin-EDTA diluted 1:2 PBS (Lonza, Verviers, Belgium) and split 1:3 in 5% collagen A-precoated T25 flasks. Small tissue fragments from gland biopsies were frozen and maintained at −80 °C for qRT-PCR experiments.

### Cell Proliferation Assay

HPG primary cell proliferation was determined using a fluorescence-based proliferation assay kit (CyQuant, Molecular Probes, Invitrogen). Briefly, 1.5 × 10 cells were seeded in 96-well plates and cultured with expansion medium. After the indicated periods of time, cells were frozen, thawed and lysed by addition of a buffer containing the green fluorescent CyQuant dye. Fluorescence was then measured using a microtiter plate reader (Tecan, Switzerland) with an excitation wavelength of 485 nm and emission detection at 530 nm.

### Immunofluorescence Microscopy

Cells were cultured in collagen A-precoated chamber slides. After culturing for 24 or 48 h, cells were fixed with 4% paraformaldehyde in PBS for 5 minutes and permeabilized with 0.2% Triton X-100 in Tris-HCl buffered saline (TBS) for 5 minutes. Samples were then washed with TBS and permeabilized twice with 0.1% Triton X-100 in TBS containing 0.1% BSA for 10 minutes each. Subsequently, cells were blocked with 10% goat serum in PBS for 30 minutes. Immunostaining was performed overnight at 4 °C in blocking solution. Secondary antibodies were incubated for 1 h at room temperature. Coverslips were mounted in Mowiol-antifade (Dako, Glostrup, Denmark) and imaged in x-y middle sections in a FluoViewTM FV1000 confocal microscope (Olympus, Center Valley, PA).

### Amylase-VAMP-2 co-distribution analysis

To quantify the co-localization of amylase and VAMP-2, circles were drawn around single fluorescent spots that were scored positive when the mean fluorescence intensity was at least three times the standard deviation of the background. Percentage co-distribution was determined in single cells and the average values were calculated from the total number of cells evaluated. In order to rule out the possibility that the observed correlation stemmed from random signal overlap, each frame was rotated 90 degrees and molecule co-distribution was again calculated.

### Electron Microscopy

Cells were cultured in chamber slides coated with 5% collagen A. After culturing for 24 or 48 h in expansion medium, and then treated with 3.5% glutaraldehyde for 40 minutes at 37 °C and washed three times with 0.1 M PBS. The inclusion was performed with 0.2% osmium for 30 minutes at room temperature in the dark. Preparations were then washed with distillated H_2_O twice and dehydrated with a series of increasing ethanol concentration. Samples were embedded in Araldite (Fluka, Buchs, Switzerland) and cut into ultrathin (50–70 nm) sections, stained with lead citrate, and examined under a Jeol JEM-1010 electron microscope (Tokyo, Japan).

### Reverse transcriptase–polymerase chain reaction (RT-PCR)

Total RNA was extracted from biopsies and HPG cell cultures using the RNAeasy Kit (Qiagen, Valencia, CA) according to the manufacturer’s instructions. One microgram of total RNA was reverse-transcribed using random hexamers and Super Script III (Invitrogen). qRT-PCR was performed using IQ SYBR Green Supermix and the human primers as follows: Aquaporin-5 (AQP5) (forward, 5′-CCTGTCCATTGGCCTGTCTGTCAC-3′; reverse, 5′-GGCTCATACGTGCCTTTGATGATG-3′), amylase (forward, 5′CTGGGTGGTGAGCCAATTAAA-3′, reverse, 5′CCACAAAGACAAGCGCTCTGT-3′), bone morphogenetic protein-6 (BMP6) (forward, 5′-CAACAGAGTCGTAATCG-3′; reverse, 5′-TTAGTGGCATCCACAAGCTCT-3′), Claudin-4 (CLDN4) (forward 5′-AGCTCTGTGGCCTCAGGACTCT-3; reverse, 5′-CAGTGATGAATAGCTCTTCTTAAATTACAA-3′), Claudin-10 (CLDN10) (forward, 5′-CTGGAAGGTGTCTACCATCGA-3′; reverse, 5′-AAAGAAGCCCAGGCTGACA-3′), ENaC subunits α-ENaC (forward, 5′-GCGACTGCACCAAGAATGGCAGTG-3′; reverse, 5′-GATGTAGGCACAGCCACACTCCTTG-3′), β-ENaC (forward, 5′-GTCAGGCTGATGCTTCACGAGCAG-3′; reverse, 5′-CTGTAGGGCTCCCCCATGCGCTG-3′), γ-ENaC (forward, 5′-GACCTGAACCAGAGATCCATCATGG-3′; reverse, 5′-CCCACCACTCCTTGGCTTTCTGCC-3′), human endogenous sequence retrovirus (hERV-3) (forward, 5′-CATGGGAAGCAAGGGAACTAATG-3′; reverse, 5′-CCCAGCGAGCAATACAGAATTT-3′).

Each primer pair was designed to span at least one exon-intron boundary. Amplification was conducted for 40 cycles with an annealing temperature of 60 °C for 60 seconds for all primers. qRT-PCR was performed using the MiniOpticon Real-Time PCR system (Biorad). The ∆∆Ct method was used for analyses^51^.

### Electrophysiological recordings

Recordings of ENaC currents were performed at room temperature using the perforated patch-clamp technique as described^24^ using an Axon 700 B amplifier and pClamp 10.0 software (Molecular Devices). Cells were perfused with extracellular saline buffer (in mM): 137NaCl, 4KCl, 1.8CaCl_2_, 1MgCl_2_, 10Glucose and 10HEPES, pH 7.4). Patch pipettes were made of borosilicate glass and had resistances of 1–3 MΩ. Intracellular solution contained (in mM): 65KCl, 45K-Gluconate, 10NaCl, 1MgCl_2_, 50Glucose and 10HEPES, pH 7.2. In some experiments extracellular NaCl was substituted with *N*-methyl-D-glucamine. Conductance-voltage relationships were obtained by measuring the amplitude of outward currents at the each depolarizing pulse from −70 mV. Data were analyzed using Igor-pro software (Wavemetrics, Lake Oswego, OR). Results are expressed as average ± SEM.

### Amylase activity assay

HPG primary cells were seeded on collagen A-precoated 96-well plates (5 × 10^4^ cells/well) with expansion medium for 24–48 h. The cells were then cultured with expansion medium alone or containing 10 mM isoproterenol plus 2 mM Ca^2+^, (enriched medium). Under these conditions cells were incubated in the presence of TNF-α, IL-1, IFN-γ, TGF-β, or CXCL12 at the indicated concentrations. After 24 h of treatment, amylase activity was assayed by a fibril-degradation assay (EnzChek Ultra Amylase Assay Kit; Molecular Probes). Fluorescence was measured with a fluorometer (Tecan), using standard fluorescein filters. Amylase concentration was calculated according to the manufacturer’s instructions. To assess the intracellular amylase concentration, cells were lysed in EnzChek Ultra Amylase Assay Kit buffer containing 2 mM of PMSF and cOmplete. Lysates were then cleared by centrifugation and 50 μL of supernatant was used for amylase assays.

### Statistical analysis

Results are expressed as the arithmetic mean ± SD of the mean, as indicated. Wilcoxon signed rank tests were used to determine significant differences and p values <0.05 were considered significant.

### Data Availability

The datasets generated during and/or analyzed during the current study are available from the corresponding author on reasonable request.

## References

[1] Fox RI (2005). Sjogren’s syndrome. Lancet.

[2] Liang Y, Yang Z, Qin B, Zhong R (2014). Primary Sjogren’s syndrome and malignancy risk: a systematic review and meta-analysis. Ann Rheum Dis.

[3] Alamanos Y (2006). Epidemiology of primary Sjogren’s syndrome in north-west Greece, 1982–2003. Rheumatology (Oxford).

[4] Kabasakal Y (2006). The prevalence of Sjogren’s syndrome in adult women. Scand J Rheumatol.

[5] Ramos-Casals M (2015). Google-driven search for big data in autoimmune geoepidemiology: analysis of 394,827 patients with systemic autoimmune diseases. Autoimmun Rev.

[6] Vitali C, Moutsopoulos HM, Bombardieri S (1994). The European Community Study Group on diagnostic criteria for Sjogren’s syndrome. Sensitivity and specificity of tests for ocular and oral involvement in Sjogren’s syndrome. Ann Rheum Dis.

[7] Shiboski SC (2012). American College of Rheumatology classification criteria for Sjogren’s syndrome: a data-driven, expert consensus approach in the Sjogren’s International Collaborative Clinical Alliance cohort. Arthritis Care Res (Hoboken).

[8] Shiboski CH (2017). 2016 American College of Rheumatology/European League Against Rheumatism Classification Criteria for Primary Sjogren’s Syndrome: A Consensus and Data-Driven Methodology Involving Three International Patient Cohorts. Arthritis Rheumatol.

[9] Fox RI, Saito I (1994). Sjogren’s syndrome: immunologic and neuroendocrine mechanisms. Adv Exp Med Biol.

[10] Manganelli P (1997). Quantitative analysis of apoptosis and bcl-2 in Sjogren’s syndrome. J Rheumatol.

[11] Humphreys-Beher MG, Brayer J, Yamachika S, Peck AB, Jonsson R (1999). An alternative perspective to the immune response in autoimmune exocrinopathy: induction of functional quiescence rather than destructive autoaggression. Scand J Immunol.

[12] Jonsson R, Kroneld U, Backman K, Magnusson B, Tarkowski A (1993). Progression of sialadenitis in Sjogren’s syndrome. Br J Rheumatol.

[13] Scott J (1980). Qualitative and quantitative observations on the histology of human labial salivary glands obtained post mortem. J Biol Buccale.

[14] Ship JA, Baum BJ (1990). Is reduced salivary flow normal in old people?. Lancet.

[15] Christodoulou MI, Kapsogeorgou EK, Moutsopoulos HM (2010). Characteristics of the minor salivary gland infiltrates in Sjogren’s syndrome. J Autoimmun.

[16] Fox RI, Kang HI, Ando D, Abrams J, Pisa E (1994). Cytokine mRNA expression in salivary gland biopsies of Sjogren’s syndrome. J Immunol.

[17] Boumba D, Skopouli FN, Moutsopoulos HM (1995). Cytokine mRNA expression in the labial salivary gland tissues from patients with primary Sjogren’s syndrome. Br J Rheumatol.

[18] Amft N (2001). Ectopic expression of the B cell-attracting chemokine BCA-1 (CXCL13) on endothelial cells and within lymphoid follicles contributes to the establishment of germinal center-like structures in Sjogren’s syndrome. Arthritis Rheum.

[19] Barone F (2005). Association of CXCL13 and CCL21 expression with the progressive organization of lymphoid-like structures in Sjogren’s syndrome. Arthritis Rheum.

[20] Kramer JM, Klimatcheva E, Rothstein TL (2013). CXCL13 is elevated in Sjogren’s syndrome in mice and humans and is implicated in disease pathogenesis. J Leukoc Biol.

[21] Moll R, Franke WW, Schiller DL, Geiger B, Krepler R (1982). The catalog of human cytokeratins: patterns of expression in normal epithelia, tumors and cultured cells. Cell.

[22] Chieregatti E, Meldolesi J (2005). Regulated exocytosis: new organelles for non-secretory purposes. Nat Rev Mol Cell Biol.

[23] Hanukoglu I, Hanukoglu A (2016). Epithelial sodium channel (ENaC) family: Phylogeny, structure-function, tissue distribution, and associated inherited diseases. Gene.

[24] Wesch D (2012). Differential N termini in epithelial Na+ channel delta-subunit isoforms modulate channel trafficking to the membrane. Am J Physiol Cell Physiol.

[25] Sakai A, Sugawara Y, Kuroishi T, Sasano T, Sugawara S (2008). Identification of IL-18 and Th17 cells in salivary glands of patients with Sjogren’s syndrome, and amplification of IL-17-mediated secretion of inflammatory cytokines from salivary gland cells by IL-18. J Immunol.

[26] Ogawa N (1995). Analysis of transforming growth factor beta and other cytokines in autoimmune exocrinopathy (Sjogren’s syndrome). J Interferon Cytokine Res.

[27] Lisi S, Sisto M, D’Amore M, Lofrumento DD, Ribatti D (2013). Emerging avenues linking inflammation, angiogenesis and Sjogren’s syndrome. Cytokine.

[28] Lisi S (2014). Chronic inflammation enhances NGF-beta/TrkA system expression via EGFR/MEK/ERK pathway activation in Sjogren’s syndrome. J Mol Med (Berl).

[29] Kagi D (1994). Fas and perforin pathways as major mechanisms of T cell-mediated cytotoxicity. Science.

[30] Abu-Helu RF, Dimitriou ID, Kapsogeorgou EK, Moutsopoulos HM, Manoussakis MN (2001). Induction of salivary gland epithelial cell injury in Sjogren’s syndrome: *in vitro* assessment of T cell-derived cytokines and Fas protein expression. J Autoimmun.

[31] Nelson J, Manzella K, Baker OJ (2013). Current cell models for bioengineering a salivary gland: a mini-review of emerging technologies. Oral Dis.

[32] Chopra DP, Xue-Hu IC (1993). Secretion of alpha-amylase in human parotid gland epithelial cell culture. J Cell Physiol.

[33] Chan YH, Huang TW, Young TH, Lou PJ (2011). Human salivary gland acinar cells spontaneously form three-dimensional structures and change the protein expression patterns. J Cell Physiol.

[34] Yin H (2013). Association of bone morphogenetic protein 6 with exocrine gland dysfunction in patients with Sjogren’s syndrome and in mice. Arthritis Rheum.

[35] Lai Z (2016). Aquaporin gene therapy corrects Sjogren’s syndrome phenotype in mice. Proc Natl Acad Sci USA.

[36] Fujita-Yoshigaki J, Matsuki-Fukushima M, Sugiya H (2008). Inhibition of Src and p38 MAP kinases suppresses the change of claudin expression induced on dedifferentiation of primary cultured parotid acinar cells. Am J Physiol Cell Physiol.

[37] Garty H, Palmer LG (1997). Epithelial sodium channels: function, structure, and regulation. Physiol Rev.

[38] Schneyer LH (1970). Amiloride inhibition of ion transport in perfused excretory duct of rat submaxillary gland. Am J Physiol.

[39] Dinudom A, Young JA, Cook DI (1993). Amiloride-sensitive Na + current in the granular duct cells of mouse mandibular glands. Pflugers Arch.

[40] McKinney JS, Rubin RP (1988). Enhancement of cyclic AMP modulated salivary amylase secretion by protein kinase C activators. Biochem Pharmacol.

[41] Putney JW (1986). Identification of cellular activation mechanisms associated with salivary secretion. Annu Rev Physiol.

[42] Vedam VK, Boaz K, Natarajan S, Ganapathy S (2016). Salivary Amylase as a Marker of Salivary Gland Function in Patients Undergoing Radiotherapy for Oral Cancer. J Clin Lab Anal,.

[43] Fujita-Yoshigaki J, Tagashira A, Yoshigaki T, Furuyama S, Sugiya H (2005). A primary culture of parotid acinar cells retaining capacity for agonists-induced amylase secretion and generation of new secretory granules. Cell Tissue Res.

[44] Bikker A (2010). Increased expression of interleukin-7 in labial salivary glands of patients with primary Sjogren’s syndrome correlates with increased inflammation. Arthritis Rheum.

[45] Ansel KM (2000). A chemokine-driven positive feedback loop organizes lymphoid follicles. Nature.

[46] Ciccia F (2015). Interleukin (IL)-22 receptor 1 is over-expressed in primary Sjogren’s syndrome and Sjogren-associated non-Hodgkin lymphomas and is regulated by IL-18. Clin Exp Immunol.

[47] Pohlers D (2009). TGF-beta and fibrosis in different organs - molecular pathway imprints. Biochim Biophys Acta.

[48] Zheng C, Hoffman MP, McMillan T, Kleinman HK, O’Connell BC (1998). Growth factor regulation of the amylase promoter in a differentiating salivary acinar cell line. J Cell Physiol.

[49] Yamamura Y (2012). TNF-alpha inhibits aquaporin 5 expression in human salivary gland acinar cells via suppression of histone H4 acetylation. J Cell Mol Med.

[50] Coric T (2004). Expression of ENaC and serum- and glucocorticoid-induced kinase 1 in the rat intestinal epithelium. Am J Physiol Gastrointest Liver Physiol.

[51] Schmittgen TD, Livak KJ (2008). Analyzing real-time PCR data by the comparative C(T) method. Nat Protoc.

